# St. George's Hospital: A Settlement in Sight

**Published:** 1914-01-24

**Authors:** 


### ST. GEORGE'S HOSPITAL.

### Steps towards a Settlement.

We are glad to be able to announce that our appeal to those responsible no longer to permit personal questions to dominate the situation has been cordially received and may prove effective for good. At a meeting of influential governors held on Tuesday, the whole position was frankly discussed. Succeeding speakers emphasised the call for unanimity and the importance to St. George's Hospital that combined action should be secured. The proceedings were harmonious and unanimous.

### An Improper Proceeding.

The whole trouble for months in connection with the affairs of St. George's Hospital has been the persistent putting forward of a personal policy and personal interests. The laws in regard to the appointment of a Medical Superintendent, for instance, have been practically suspended—a most improper proceeding. It is pleasant therefore to be able to record that the acting members of the medical staff, in November 1913, intimated to the Sub-Committee to which the question of appointing a Medical Superintendent has been referred, more than once, that the Medical Staff Committee advised the Sub-Committee of the House Committee then sitting to recommend that the post of Resident Medical Superintendent be advertised. Not only was this advice disregarded, but as the proceedings at the Special Court on December 19 proved, a proposal was put forward to appoint one of the treasurers Secretary-Superintendent, at a large salary. Notwithstanding the rejection of this proposal at the Special Court by a majority of something like two votes to one, no steps were taken at subsequent House Committees to advertise for a Medical Superintendent, but, on the contrary, the question of this appointment was once again referred to a Sub-Committee, a step which could not be regarded as in the interests of the hospital, by any impartial person with a knowledge of the facts.

### Justice for the Acting Medical Staff.

It is only just that the public and the profession should know that the Medical Staff Committee have been set aside and their advice disregarded, a proceeding which has not contributed to the efficient administration of the hospital. The active members of the staff naturally desire to keep the whole of the hospital work efficient and up to date, hence no doubt their advice and persistent attempts to secure the immediate appointment of a Medical Superintendent. One of the consulting surgeons has taken and promoted the opposite view to that represented by the advice referred to, which has not unnaturally given rise to the impression that the staff have supported Mr. West continuously and collectively, which is not a fact. It is due to the acting medical staff that we should make these points perfectly clear. The medical staff, to their honour, have now succeeded in getting the appointment for a Medical Superintendent advertised.

### The Responsibility of High Office.

There are some dangers which never affect a voluntary hospital where the true spirit of personal service dominates everyone holding high office. In such circumstances all personal considerations and views are properly subordinated by high officials to the interests of their hospital. Unfortunately this spirit appears to have been sadly absent from much which has happened at St. George's Hospital during the past seven months at any rate. We have good reason to hope that the causes which have created this unsatisfactory state of affairs are about to be removed. It would, moreover, be disastrous if merely personal considerations, backed by bad advice and an absence of accurate information, should create an effort to foist the mistakes and bad policy of the past upon some who have properly and wisely not yet intervened. Prejudice and bad advisers are the danger and sometimes the destruction of monarchs. Weak policies, founded upon personal aims, or dependent upon personal ends, must ultimately destroy a Government which supports them. It is therefore to be hoped before any final step is taken in the matter of St. George's Hospital by independent officers who have the confidence and support of the governors, that they will put themselves in a position to learn all the facts through an impartial channel, and then determine upon their course of action.

### The Amalgamation with the Westminster Hospital.

Nothing should now delay the steady advancement to completion of the negotiations for amalgamation between the Westminster and St. George's Hospitals. It will be remembered that at the meeting of the Special Court of Governors of St. George's Hospital on the 19th ultimo, the governors withheld their consent from a proposal put forward at that meeting to purchase the Wandsworth Bridge site for £27,000, on the ground that, as trustees, the governors could not go further than give authority to the House Committee to obtain an option upon it at their discretion. The governors felt they could not be a party to the purchase of a site which might land the hospital in a heavy loss on re-sale. The wisdom of this decision on the part of the governors has been amply confirmed by the fact that at a subsequent meeting of the House Committee, when a renewed offer to sell this site came up for consideration, it was resolved, as we are advised, not to accept the offer. The ground is therefore open for a renewal of the conferences between the hospitals with a view to amalgamation.

---

*Truth* for 21st January contains an article entitled, "The West affair at St. George's."

